# Lipoteichoic acid of *Streptococcus gordonii* as a negative regulator of human dendritic cell activation

**DOI:** 10.3389/fimmu.2023.1056949

**Published:** 2023-03-28

**Authors:** Sun Kyung Kim, Jintaek Im, Eun Byeol Ko, Dongwook Lee, Ho Seong Seo, Cheol-Heui Yun, Seung Hyun Han

**Affiliations:** ^1^ Department of Oral Microbiology and Immunology, and Dental Research Institute, School of Dentistry, Seoul National University, Seoul, Republic of Korea; ^2^ Research Division for Biotechnology, Korea Atomic Energy Research Institute, Jeongeup, Republic of Korea; ^3^ Department of Agricultural Biotechnology, and Research Institute of Agriculture and Life Sciences, Seoul National University, Seoul, Republic of Korea; ^4^ Institutes of Green-bio Science and Technology, Seoul National University, Pyeongchang, Gangwon-do, Republic of Korea; ^5^ Interdisciplinary Programs in Agricultural Genomics, Seoul National University, Seoul, Republic of Korea

**Keywords:** dendritic cell, immune evasion, lipoteichoic acid, lipoprotein, *Streptococcus gordonii*, T cell activation

## Abstract

*Streptococcus gordonii*, an opportunistic Gram-positive bacterium, causes an infective endocarditis that could be fatal to human health. Dendritic cells (DCs) are known to be involved in disease progression and immune responses in *S. gordonii* infection. Since lipoteichoic acid (LTA) is a representative virulence factor of *S. gordonii*, we here investigated its role in the activation of human DCs stimulated with LTA-deficient (Δ*ltaS*) *S. gordonii* or *S. gordonii* LTA. DCs were differentiated from human blood-derived monocytes in the presence of GM-CSF and IL-4 for 6 days. DCs treated with heat-killed Δ*ltaS S. gordonii* (Δ*ltaS* HKSG) showed relatively higher binding and phagocytic activities than those treated with heat-killed wild-type *S. gordonii* (wild-type HKSG). Furthermore, Δ*ltaS* HKSG was superior to wild-type HKSG in inducing phenotypic maturation markers including CD80, CD83, CD86, PD-L1, and PD-L2, antigen-presenting molecule MHC class II, and proinflammatory cytokines such as TNF-α and IL-6. Concomitantly, DCs treated with the Δ*ltaS* HKSG induced better T cell activities, including proliferation and activation marker (CD25) expression, than those treated with the wild-type. LTA, but not lipoproteins, isolated from *S. gordonii* weakly activated TLR2 and barely affected the expression of phenotypic maturation markers or cytokines in DCs. Collectively, these results demonstrated that LTA is not a major immuno-stimulating agent of *S. gordonii* but rather it interferes with bacteria-induced DC maturation, suggesting its potential role in immune evasion.

## Introduction

*Streptococcus gordonii* is a Gram-positive bacterium found primarily in various mucosal tissues, such as the oral cavity, gut, and skin in human (1). *S. gordonii* is considered as an opportunistic pathogen associated with the pathogenesis of various infectious diseases. For example, *S. gordonii* acts as an initial colonizer during development of dental plaque in human (2) that is closely related to the pathogenicity of dental caries and apical periodontitis (3). Furthermore, it causes life-threatening systemic diseases such as septic arthritis (4) and infective endocarditis (5). Moreover, *S. gordonii* can directly induce inflammatory responses by using serine-rich repeat adhesins that bind to and activate host cells (6). On the other hand, *S. gordonii* can efficiently evade the surveillance of the host immune system by producing various factors such as peptidylprolyl *cis*-*trans* isomerase and a lipoprotein which can exacerbate infective endocarditis through inhibiting phagocytosis (7).

Lipoteichoic acid (LTA) is regarded as a representative cell-wall virulence factor derived from Gram-positive bacteria and is involved in bacterial division, autolysis and survival (8, 9). Although LTA has common structural motif and function in most Gram-positive bacteria (10), its immunostimulatory potency seems to be heterogeneous among bacterial species. LTAs purified from pathogens such as *Staphylococcus aureus* and *Streptococcus pneumoniae* tend to induce proinflammatory molecules, including tumor necrosis factor (TNF)-α and prostaglandin E2 *via* Toll-like receptor (TLR) 2 dependent manner in phagocytes (11, 12). Combined treatment with *S. aureus* LTA and muramyl dipeptide substantially elicited the human monocyte-derived dendritic cells (DCs) maturation and activation (13). In addition, LTA isolated from *Lacticaseibacillus rhamnosus* GG promoted the maturation and cytokine expression of mouse bone marrow-derived DCs *in vitro* and its oral administration increased the population of CD103^+^ DC in the Peyer’s patches (14). In contrast, LTA from *Lactobacillus plantarum*, one of the commensal bacteria, produced anti-inflammatory effects (15, 16) and protected the host from endotoxin-induced septic shock (17). Moreover, it has been reported that LTA from *Staphylococcus epidermidis* suppressed both inflammatory cytokine released from keratinocytes and inflammation in rodent skin injury model (18). On the other hand, little is known about the immunomodulatory effects of LTA from *S. gordonii*, which can cause opportunistic infections (1).

DCs are known as professional antigen-presenting cells that not only recognize but also capture invading pathogens and present microbial antigens to T cells, activating antigen-specific adaptive immunity (19). Indeed, DCs express high levels of pattern-recognition receptors to sense and respond to microbe-associated molecular patterns (MAMPs), such as LTA, peptidoglycan, and lipoproteins (20). Upon the recognition of MAMPs, DCs upregulate the expression of costimulatory molecules, including cluster of differentiation (CD) 80, CD86, and CD40, and major histocompatibility complex (MHC) class II (13, 21). In addition, they induce cytokines (22) that are pivotal for not only differentiation but also activation of effector T cells (19). Of note, DCs are importantly involved in *S. gordonii*-caused infectious diseases through promoting the differentiation of monocytes to DCs and stimulating proinflammatory cytokines (1, 23, 24). Therefore, we here examined whether LTA affects the phenotypic and functional activation of DCs in response to *S. gordonii* with the wild-type bacteria, its LTA-deficient strain (Δ*ltaS*), or LTA isolated from *S. gordonii*.

## Materials and methods

### Reagents and chemicals

RPMI 1640, fetal bovine serum (FBS), and penicillin-streptomycin solutions were acquired from HyClone (Logan, UT, USA). Bacto™ Todd Hewitt (TH) broth, BBL™ yeast extract, anti-human CD14- and CD3-magnetic particles, and BD IMag™ Cell Separation Magnet were acquired from BD Biosciences (San Diego, CA, USA). Fluorescein isothiocyanate (FITC)-conjugated anti-human-CD80 antibody, phycoerythrin (PE)-conjugated anti-human-CD4, -CD8, -CD25, -CD83, and -PD-L2 antibodies, allophycocyanin (APC)-conjugated anti-human-CD86, and -programmed death-ligand 1 (PD-L1) antibodies, and their isotype control antibodies were also acquired from BioLegend (San Diego, CA, USA). FITC-labeled anti-human MHC class II antibody together with its isotype control antibody were acquired from BD Biosciences. All kits for enzyme-linked immunosorbent assay (ELISA) used in the quantification of cytokine productions were acquired from BioLegend.

### Preparation of human DCs

Peripheral blood mononuclear cells (PBMCs) were isolated from human blood samples, supplied from the Korean Red Cross (Seoul, Republic of Korea), by density-gradient centrifugation with Ficoll (GE Healthcare, Uppsala, Sweden). Then, CD14^+^ monocytes were purified from PBMCs using anti-human CD14 magnetic particles and BD IMag™ Cell Separation Magnet. The cells were differentiated into DCs for 6 days in RPMI 1640 medium containing 10% FBS, 1% penicillin-streptomycin solution, human recombinant granulocyte-macrophage colony-stimulating factor (GM-CSF; 5 ng/ml; R&D Systems, Minneapolis, MN, USA), and interleukin-4 (IL-4) (9 ng/ml; CreaGene, Gyeonggi-Do, Republic of Korea) (23).

### Generation of LTA-deficient *S. gordonii*

The *S. gordonii* CH1 wild-type strain was kindly provided by Prof. Paul Sullam at the University of California, San Francisco (San Francisco, CA, USA). The *S. gordonii* CH1-derived, LTA-deficient strain (Δ*ltaS*) was generated as previously described (25). Briefly, a gene replacement vector (pC-Δ*ltaS*) was constructed by cloning the flanking upstream and downstream regions of *ltaS* into a suicide plasmid (pC326). The upstream flanking region was amplified and digested with *Kpn*I (New England Biolabs, Ipswich, MA, USA) and *Xho*I (New England Biolabs). The downstream flanking region was amplified, followed by digestion with *Bam*HI (New England Biolabs) and *Not*I (New England Biolabs). Then, the downstream polymerase chain reaction products were cloned into the pC326 and introduced into *S. gordonii* CH1 strain by natural transformation.

### Preparation of heat-inactivated bacteria

Heat-killed *S. gordonii* (HKSG) was prepared for wild-type (wild-type HKSG) and Δ*ltaS* (Δ*ltaS* HKSG) strains. Briefly, the wild-type and Δ*ltaS S. gordonii* were cultured in TH medium supplemented with 0.5% yeast extract (THY) at 37°C until the mid-exponential phase and collected by centrifuging at 8,944 × *g* for 10 min. The resuspended bacterial pellet in phosphate-buffered saline (PBS) was inactivated at 70°C for 1 h. Inactivation of bacteria was confirmed by observing no bacterial growth on THY agar plates (*data not shown*).

### Preparation of LTAs

LTA was purified from *S. gordonii* CH1 (Sg.LTA) as described previously (26). Briefly, bacteria pellet disrupted by ultrasonication in 0.1 M sodium citrate buffer was subjected to organic solvent extraction using *n*-butanol (Junsei Chemical Co., Tokyo, Japan). Next, dialysis was conducted for the aqueous phase using dialysis membrane (Spectra/Por 6; Spectrum^®^ Laboratories Inc., Ranch Dominquez, CA, USA) in endotoxin-free water (Dai Han Pharm Co. Ltd., Seoul, Republic of Korea). Hydrophobic interaction chromatography with Octyl-Sepharose CL-4B (Sigma-Aldrich, St. Louis, MO, USA) and ion-exchange chromatography with diethylaminoethyl-sepharose (Sigma-Aldrich) were sequentially performed to obtain highly-pure LTA. The LTA was quantified by measuring its dry weight as previously described (12). The LTA had no detectable amount of any biological contaminants (data not shown).

### Preparation of *S. gordonii* lipoproteins

Lipoprotein was purified from *S. gordonii* CH1 (Sg.LPP) as previously described (25). Briefly, a bacterial pellet was suspended in Tris-buffered saline (TBS) containing protease inhibitors. The bacterial cells were disrupted by ultrasonication and mixed with 20% Triton X-114 at 9:1 ratio. The supernatants collected by centrifugation were mixed with the same amount of TBS and incubated at 37°C for additional 15 min. After the overnight incubation of mixture in methanol at -20°C, pellet was collected by centrifugation and solubilized in 10 mM octyl β-d-glucopyranoside solution. The Sg.LPP was then quantified by measuring its dry weight after lyophilization.

### Analysis of DC phagocytic capacity

To label HKSG with carboxyfluorescein succinimidyl ester (CFSE), wild-type and Δ*ltaS* HKSGs were incubated in 1 ml PBS containing 10 µM carboxyfluorescein diacetate succinimidyl ester (CFDA-SE, Molecular Probes, Eugene, OR, USA) for 15 min at 37°C. After washing with PBS three times, the number of bacteria was adjusted to 1 × 10^10^ CFU/ml in PBS. The DCs (5 × 10^4^ cells) were then incubated with 1.5 × 10^6^ or 5 × 10^6^ CFU of HKSG labeled with CFSE at 4°C or 37°C for 1 h. After washing the cells with PBS, bacterial adherence to DCs at 4°C and bacterial internalization into DCs at 37°C were measured by using flow cytometry (FACSCalibur, BD Biosciences). Phagocytosis was calculated by subtracting the percentage of CFSE^+^ cells at 4°C from the percentage of CFSE^+^ cells at 37°C. All flow cytometric data in the current study were analyzed by FlowJo software (TreeStar, San Carlos, CA, USA).

### Analysis of DC viability

In the presence of GM-CSF (2.5 ng/ml) and IL-4 (4.5 ng/ml), DCs (2.5 × 10^5^ cells/ml) treated with wild-type or Δ*ltaS* HKSG (10^6^, 10^7^ or 10^8^ CFU/ml) for 24 h were incubated with propidium iodide (PI) (Sigma-Aldrich) and Annexin V conjugated with FITC (BioLegend) for 10 min. Cell viability was then measured using flow cytometry (FACSCalibur) (27).

### Phenotypic analysis of DCs

The DCs (2.5 × 10^5^ cells/ml) were treated with wild-type or Δ*ltaS* HKSG (10^6^, 10^7^, and 10^8^ CFU/ml) or Sg.LTA or Sg.LPP at 10 μg/ml in the presence of GM-CSF and IL-4 for 24 h. The cells were incubated with fluorochrome-conjugated antibodies specific for anti-human CD80, CD83, CD86, MHC class II, PD-L1, and PD-L2 at 4°C for 30 min. The geometric mean fluorescence intensity (MFI) was measured by flow cytometry (FACSCalibur).

### Cytokine quantification

The culture supernatants were collected from DCs (2.5 × 10^5^ cells/ml) treated with wild-type or Δ*ltaS* HKSG (1 × 10^6^, 1 × 10^7^, and 1 × 10^8^ CFU/ml) or Sg.LTA or Sg.LPP at 10 μg/ml in the presence of GM-CSF and IL-4 for 24 h. Then, cytokine productions were quantified by ELISA.

### Transfection and reporter gene assay

HEK293 cells overexpressing human TLR2 (293-hTLR2; *In vivo*Gen, San Diego, CA, USA) were maintained in Dulbecco’s modified eagle medium containing 10% FBS, 1% penicillin-streptomycin solution, and 10 μg/ml blasticidin (*In vivo*Gen). The 293-hTLR2 cells (5 × 10^5^ cells/ml) were transfected with NF-κB luciferase reporter construct (Clontech Laboratories, Mountain View, CA, USA) using Attractene transfection reagent (QIAGEN^®^, Venlo, Netherlands). After stimulating the transfected cells with either 1 × 10^7^ and 1 × 10^8^ CFU/ml of wild-type or Δ*ltaS* HKSG for 20 h, the cell lysate was prepared with Passive Lysis Buffer (Promega, Madison, WI, USA). Then, Bright-Glo™ substrate (Promega) was added to the cell lysate to quantify the luciferase activity with GloMax^®^ 96 Microplate Luminometer (Promega).

### Analysis of T cell-stimulating activity of DCs

After CD14^+^ cell isolation from PBMCs for DC preparation, autologous CD3^+^ T cells were also isolated using anti-human CD3 magnetic particles, and BD IMag™ Cell Separation Magnet. Then, the CD3^+^ T cells were labeled with CFSE by the addition of CFDA-SE (10 μM) containing RPMI 1640 medium for 15 min at 37°C. The DCs (2.5 × 10^5^ cells/ml) treated with wild-type or Δ*ltaS* HKSG (1 × 10^7^ CFU/ml) for 16 h, were co-cultured with the CFSE-labeled CD3^+^ T cells at 1:1 ratio for 4 days. For T cell proliferation analysis, the co-cultured cells were applied to flow cytometry (FACSCalibur). For the T cell activation analysis, the cells incubated with anti-human CD25 antibody or anti-human CD4 and CD8 antibodies were subjected to flow cytometry (FACSCalibur).

### TLR2-mediated NF-κB activation by LTA and LPP

NF-κB activation mediated by TLR2 was analyzed with CHO/CD14/TLR2 reporter cells as previously described (28, 29). After treatment with 0-10 μg/ml of Sg.LTA or Sg.LPP for 20 h, the cells were incubated with anti-human CD25 antibody and then subjected to flow cytometry (FACSCalibur).

### Statistical analysis

Results are indicated as the mean ± standard error of the mean (SEM). The normality of data was analyzed by Kolmogorov-Smirnov test using GraphPad Prism 6 software, and all the obtained data were normally distributed. The statistical significance was analyzed by one-way ANOVA with *post hoc* Tukey’s multiple comparisons test using GraphPad Prism 6 software. *P* values below 0.05 between the experimental groups or the experimental group and the control group were considered statistically significant.

## Results

### *ΔltaS* HKSG displays enhanced adherence to and internalization into DCs compared with the wild-type HKSG

**Figure 1:**
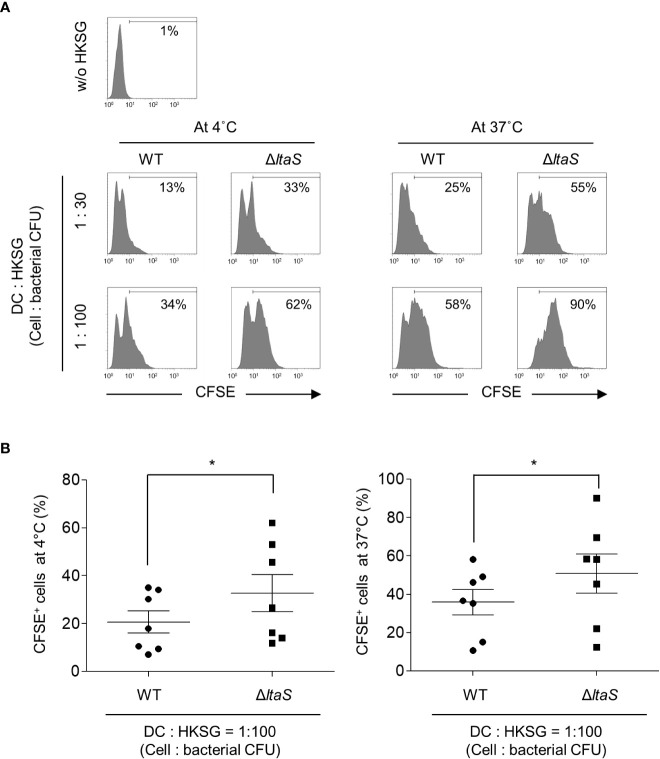
*ΔltaS* HKSG displays enhanced adherence and internalization into DCs, compared with the wild-type HKSG. DCs were prepared and treated with CFSE-labeled wild-type or Δ*ltaS* HKSG at 4°C or 37°C for 1 h **(A)** Bacterial adherence to DCs at 4°C and phagocytosis by DCs at 37°C were evaluated by flow cytometric analysis. The number on the histogram indicates the percentage of CFSE^+^ cells. **(B)** Under the same condition, mean percentage of CFSE^+^ cells indicating bacterial binding at 4°C and 37°C ± SEM from seven independent experiments is presented in scatterplots. *P < 0.05.

During microbial infection, DCs initially capture and internalize microbes to process and present microbial antigens to T cells. In addition, bacterial binding and uptake are important processes for DC maturation (30, 31). To determine the role of LTA in the binding and internalization of *S. gordonii* into DCs, CFSE-labeled wild-type or Δ*ltaS* HKSG was treated to human monocyte-derived DCs at 4°C or 37°C and the binding and internalization of HKSG were examined. At 4°C, DCs exhibited enhanced binding capacity to Δ*ltaS* HKSG compared with wild-type HKSG (**Figures 1A, B**; *left*). In addition, DCs phagocytosed Δ*ltaS* HKSG more efficiently than wild-type HKSG (**Figures 1A, B**; *right*). These results suggest that Sg.LTA interferes with the binding and internalization of *S. gordonii* to DCs.

### *ΔltaS* HKSG induces the expression of phenotypic markers of DC maturation more potently than the wild-type HKSG

**Figure 2:**
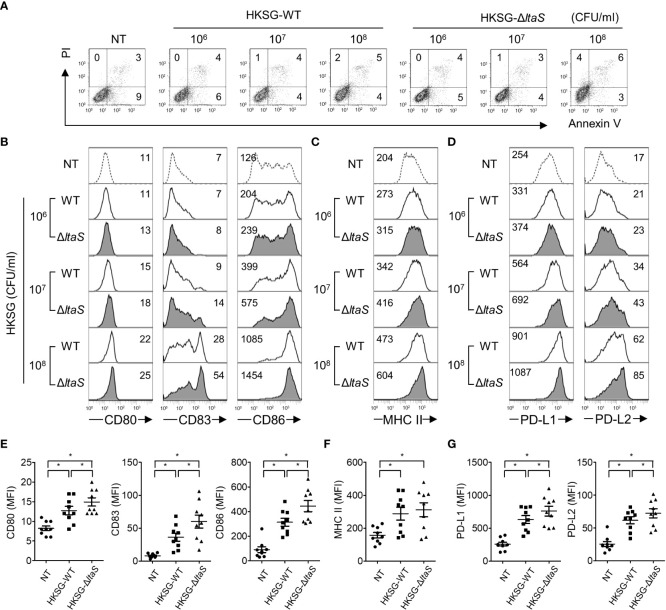
*ΔltaS* HKSG more potently upregulates the expression of phenotypic markers of DC than the wild-type HKSG did. Human DCs were treated with wild-type or Δ*ltaS* HKSG for 24 h **(A)** The untreated and HKSG-treated DCs were stained with PI and FITC-conjugated Annexin V, and then cell viability was analyzed by flow cytometry. **(B–D)** Flow cytometry was used to analyze expressions of **(B)** CD80, CD83, and CD86, **(C)** MHC class II, and **(D)** PD-L1 and PD-L2. The numbers on the histogram represent the MFI of the DCs. **(E–G)** The mean MFI of DCs treated with HKSG (1 × 10^7^ CFU/ml) ± SEM from nine independent experiments for each molecule are indicated in scatterplots. *P < 0.05.

DC maturation, a pivotal process needed for acquiring adaptive immunity, is accompanied by the upregulated expression of costimulatory molecules, MHC class II, and appropriate cytokines (13). To evaluate the action of Sg.LTA on *S. gordonii*-induced phenotypic maturation, DCs were treated with wild-type or Δ*ltaS* HKSG followed by analysis of the expression of costimulatory molecules. As shown in **Figure 2A**, stimulation with wild-type or Δ*ltaS* HKSG did not affect the viability of DCs. DCs treated with Δ*ltaS* HKSG expressed higher levels of CD80, CD83, and CD86 on their surfaces than those treated with the wild-type HKSG (**Figures 2B, E**). On the other hand, the wild-type and Δ*ltaS* HKSG exhibited similar potencies in the induction of MHC class II on DCs (**Figures 2C, F**). In addition, DCs treated with Δ*ltaS* HKSG expressed PD-L1 and PD-L2 higher than those treated with the wild-type HKSG (**Figures 2D, G**). These results indicate that Sg.LTA could act as a suppressor of phenotypic maturation of DCs induced by *S. gordonii*.

### *ΔltaS* HKSG more potently induces proinflammatory cytokines in DCs and TLR2 activation than the wild-type HKSG

**Figure 3:**
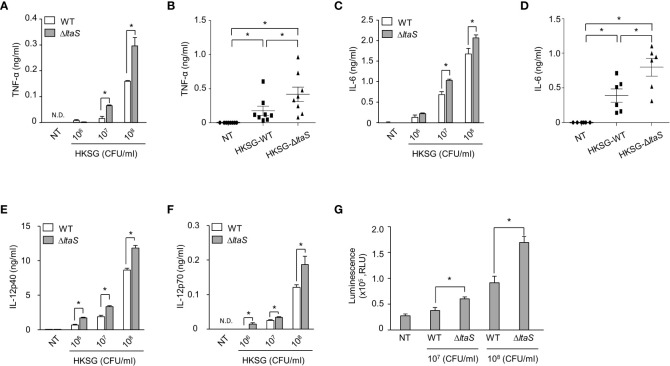
*ΔltaS* HKSG induces inflammatory cytokines in DCs and TLR2 activation more potently than the wild-type HKSG did. **(A, C, E, F)** Human DCs were treated with wild-type or Δ*ltaS* HKSG for 24 h, and then the production of **(A)** TNF-α, **(C)** IL-6, **(E)** IL-12p40, and **(F)** IL-12p70 was measured in the cell culture supernatant by ELISA. Cytokine levels are represented in the mean ± SEM of triplicates from an experiment. *P < 0.05. N.D. denotes ‘not detected.’ **(B, D)** Human DCs were treated with wild-type or Δ*ltaS* HKSG at 1 × 10^7^ CFU/ml for 24 h and cytokine levels were measured in the cell culture supernatant by ELISA. The mean concentration of TNF-α and IL-6 ± SEM from eight and six independent experiments, respectively, are indicated in scatterplots. *P < 0.05. **(G)** The 293-hTLR2 cells transfected with NF-κB luciferase reporter construct were treated with wild-type or Δ*ltaS* HKSG for 20 h, and the transcriptional activity of NF-κB was analyzed. Values are represented in the mean ± SEM of triplicates from an experiment. Values are meant *P < 0.05.

Once activated, DCs produce cytokines such as TNF-α, IL-6 and IL-12 and regulate the differentiation and activation of T cells (32). To examine the role of Sg.LTA in HKSG-induced proinflammatory cytokine production, DCs were incubated with wild-type or Δ*ltaS* HKSG, and then the cytokine production was measured. DCs treated with Δ*ltaS* HKSG exhibited enhanced production of TNF-α compared with those treated with the wild-type HKSG (**Figures 3A, B**). Consistently, Δ*ltaS* HKSG more potently induced IL-6 production than the wild-type HKSG did (**Figures 3C, D**). On the other hand, we also found that DCs treated with Δ*ltaS* HKSG showed relatively higher IL-12p40 and IL-12p70 production than those treated with the wild-type HKSG (**Figures 3E, F**). Because all tested cytokines are produced in a NF-κB-dependent manner (33, 34), we compared the NF-κB-activating capacities of the wild-type and Δ*ltaS* HKSG using a reporter gene assay in 293-hTLR2 cells. Δ*ltaS* HKSG more potently enhanced the transcriptional activity of NF-κB than the wild-type HKSG did (**Figure 3G**). These results demonstrated that Sg.LTA inhibits the *S. gordonii*-induced expression of proinflammatory cytokines by DCs.

### DCs sensitized with *ΔltaS* HKSG induce autologous T cell proliferation and activation more potently than those sensitized with the wild-type HKSG

**Figure 4:**
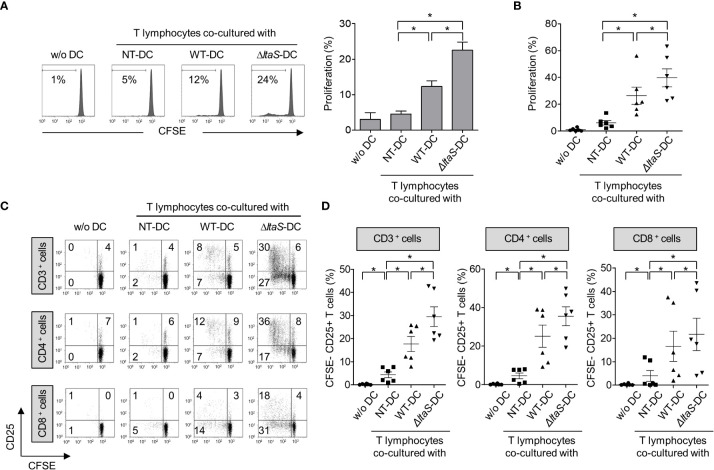
DCs sensitized with Δ*ltaS* HKSG induce the T cell proliferation and activation more potently than those sensitized with the wild-type HKSG. Human DCs were treated with wild-type or Δ*ltaS* HKSG cells for 16 h. The untreated or HKSG-treated DCs were co-cultured with CFSE-labeled autologous CD3^+^ T cells for 4 days. **(A)** Proliferation of CD3^+^ T cells was analyzed by flow cytometry. The experimental data were presented by the histogram of a representative (*left*) or the mean ± SEM of triplicates from an experiment (*right*). *P < 0.05. **(B)** The mean percentage of proliferated CD3^+^ T cells ± SEM from six separated experiments is indicated in a scatterplot. **(C)** The proliferative ability and CD25 expression of CD3^+^, CD4^+^, and CD8^+^ T cells were examined by flow cytometry. **(D)** Scatterplots show the mean frequencies of CD3^+^, CD4^+^, and CD8^+^ T cells activated by DCs ± SEM from six independent experiments.

Mature DCs can efficiently activate T cells (19). Thus, in this experiment, we compared the ability of DCs pulsed with wild-type or Δ*ltaS* HKSG for induction of autologous T cell proliferation and activation. As shown in **Figures 4A, B**, DCs sensitized with Δ*ltaS* HKSG more potently induced proliferation of T cells than those sensitized with the wild-type HKSG. Notably, Δ*ltaS*-treated DCs induced the proliferation of both CD4^+^ and CD8^+^ T cells. Consistently, DCs treated with Δ*ltaS* HKSG more efficiently induced the expression of T cell activation marker CD25 on both CD4^+^ and CD8^+^ T cells than those sensitized with the wild-type HKSG (**Figures 4C, D**). Furthermore, DCs treated with Δ*ltaS* HKSG more potently enhanced T cell activation marker CD69 and interferon-γ (IFN-γ) expression in both CD4^+^ and CD8^+^ T cells as well as IL-4 expression in CD4^+^ T cells than those treated with wild-type HKSG did (**Supplementary Figure 1**). Therefore, the Sg.LTA appears to attenuate the autologous T cellactivation ability of DCs.

### Phenotypic maturation and cytokine production of DCs are more potently induced by Sg.LPP than by Sg.LTA

**Figure 5:**
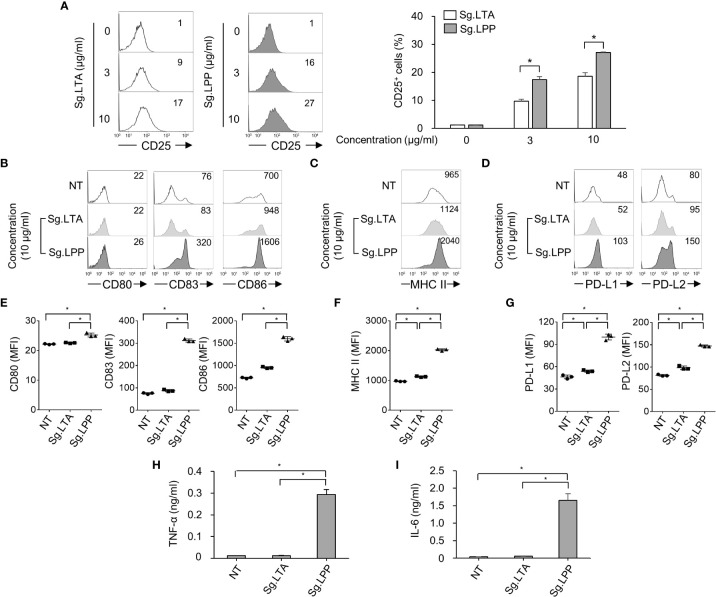
Phenotypic maturation and cytokine production of DCs are mainly stimulated by *S. gordonii* LPP rather than LTA. **(A)** CHO/CD14/TLR2 cells were treated with Sg.LTA or Sg.LPP for 20 h and then subjected to flow cytometry to measure CD25 expression. The numbers on the histogram indicate the percentages of CD25^+^ cells. The experimental data were presented by the histogram of a representative (*left*) or the mean ± SEM of triplicates from an experiment (*right*). **(B-D)** Human DCs were treated with Sg.LTA or Sg.LPP for 24 h. Then, the expression of **(B)** CD80, CD83, and CD86, **(C)** MHC class II, and **(D)** PD-L1 and PD-L2, was determined by flow cytometry. The numbers on the histogram indicate the MFI. **(E-G)** The mean MFI of DCs treated with Sg.LTA or Sg.LPP (10 μg/ml) ± SEM from three independent experiments for each molecule are indicated in scatterplots. **(H, I)** Human DCs were treated with Sg.LTA or Sg.LPP for 24 h and the production of **(H)** TNF-α and **(I)** IL-6 in the cell culture supernatant was measured using ELISA. The cytokine levels are shown as the mean ± SEM of triplicates from an experiment. *P < 0.05.

Furthermore, we examined the direct effect of Sg.LTA on DC maturation marker, antigen-presenting molecule, and cytokine expression. In addition, since the LTA may be possibly contaminated by LPP during the LTA purification (35), we confirmed that Sg.LTA used in the current study is highly-pure without contamination by Sg.LPP through silver staining and Western blot analysis (**Supplementary Figures 2A, B**). Because both LTA and LPP are commonly sensed by TLR2 (36, 37), we initially examined TLR2-mediated NF-κB activation upon exposure to Sg.LTA and Sg.LPP using CHO/CD14/TLR2 cells. Although CD25 expression was enhanced by both Sg.LTA and Sg.LPP, Sg.LPP induced significantly higher CD25 expression than Sg.LTA did (**Figure 5A**). Furthermore, the possibility of Sg.LPP contamination in the purified Sg.LTA was examined. Neither proteinase K nor lipoprotein lipase treatment to inactivate the residual Sg.LPP affected the TLR2-stimulating activity of Sg.LTA, implying that the contamination of Sg.LPP in the purified Sg.LTA is not likely (**Supplementary Figure 2C**). To estimate direct effect of Sg.LTA on the DC maturation and activation, DCs were treated with either Sg.LTA or Sg.LPP and then markers for DC maturation and antigen presentation were examined. As shown in **Figures 5B–G**, DCs treated with Sg.LTA rarely induced expression of the phenotypic markers including CD80, CD83, CD86, PD-L1, and PD-L2, and antigen-presenting molecule MHC class II. However, Sg.LPP dramatically augmented the expression of all the aforementioned molecules. Under the same condition, Sg.LTA hardly induced the productions of TNF-α and IL-6 while Sg.LPP substantially induced those cytokines (**Figures 5H, I**). In addition, we tested the effects of LTAs isolated from *Streptococcus mutans* (Sm.LTA) and *S. aureus* (Sa.LTA) on DC maturation and activation. Like Sg.LTA, other LTAs hardly affected DC maturation and activation even though Sa.LTA marginally did it (**Supplementary Figure 3**). These results demonstrated that Sg.LTA barely affects DC maturation and cytokine expression, whereas Sg.LPP is a major cellwall component responsible for DC maturation and cytokine expression.

## Discussion

*S. gordonii* can cause a life-threatening systemic disease whose diagnosis and treatment are difficult. Although DCs are crucial for *S. gordonii*-caused infectious disease progression and immune responses against it, only a few etiologic agents such as serine-rich repeat adhesins have been identified for inducing DC maturation and activation (1, 23, 24). In the current study, we have demonstrated the negative effects of Sg.LTA in the *S. gordonii*-induced human DC maturation and function. Furthermore, such effect appears to change subsequent adaptive immunity because Δ*ltaS* HKSG-sensitized DCs more potently enhanced autologous T cell proliferation and activation than the wild-type HKSG-sensitized DCs did. Of note, Sg.LTA is a weak TLR2 ligand and barely affected DC maturation marker or cytokine expression, whereas Sg.LPP, a strong TLR2 ligand, potently induced maturation and activation of DCs. Collectively, our results suggest that *S. gordonii* might utilize its LTA for immune evasion by suppressing DC maturation and activation and consequently interfering with the induction of appropriate immune responses.

We demonstrated that the Δ*ltaS* HKSG induces the maturation and cytokine expression of DCs more potently than the wild-type HKSG did. The current findings are coincident with the previous report (25) that Δ*ltaS* more potently induced proinflammatory cytokines from macrophages. Moreover, Δ*ltaS* more potently induced nitric oxide (NO) (38) and IL-8 expression than the wild-type strain in human periodontal ligament cells (39). Unlike Sg.LTA, Sg.LPP is supposed to be a potent immuno-stimulating agent of Gram-positive bacteria because Sg.LPP remarkably induced DC maturation marker expressions in this study. In keeping with our observation, LPP-deficient *S. gordonii* induced less proinflammatory cytokine and NO production than the wild-type did (25, 38). Moreover, LPP-deficient *S. aureus* failed to induce osteoclast activation and differentiation (40). Therefore, Sg.LTA and Sg.LPP might be in a competing relationship, at least in the activation of innate immune responses.

The Δ*ltaS* HKSG, in comparison with the wild-type HKSG, displayed enhanced binding/internalization to DCs in the current study. Since bacterial binding/internalization has a positive correlation with the maturation and activation of DCs (30, 31), the enhanced binding/internalization of Δ*ltaS* HKSG may induce, at least in part, an increased maturation and activation of DCs. Notably, *S. gordonii* utilizes its surface molecules such as serine-rich repeat adhesins and antigen I/II family polypeptides for binding to host cells (41, 42) and *ΔltaS S. gordonii* is known to express more abundant antigen I/II family polypeptides such as SspA and SspB (43). On the other hand, exogenous treatment with LTA isolated from *Streptococcus faecalis* suppressed expression of integrin in human urothelial cells (44), which is a host surface protein required for the cellular uptake of bacteria (45). Therefore, Sg.LTA might negatively regulate surface molecules of *S. gordonii* and/or DCs that are required for the bacterial binding/internalization and the enhanced binding/internalization of *ΔltaS S. gordonii* to DCs might lead to increased DC activation and function.

Although the current study has demonstrated that Sg.LTA exhibits an inhibitory effect on DC maturation and activation, we should yet further characterize the exact underlying mechanism at molecular level. Because the purified Sg.LPP strongly induced the maturation and cytokine production of DCs, we propose that Sg.LTA might interfere with the Sg.LPP-induced DC maturation and activation mechanism. This postulation could be explained by the following potential mechanism. Firstly, because both Sg.LTA and Sg.LPP use TLR2 for their recognition, Sg.LTA could compete with or block the TLR2 binding of Sg.LPP. Secondly, Sg.LTA could use TLR co-receptor(s) such as CD36 to weaken the TLR2 signaling pathway. Although CD36 mediates the NF-κB activation for DC maturation and activation, it could conversely inactivate NF-κB by the activation of peroxisome proliferator-activated receptor-γ (PPAR-γ) (46). Indeed, LTA was reported to interact with CD36 (47) and consequently activate PPAR-γ (48). Thus, Sg.LTA may inhibit the Sg.LPP-mediated DC maturation and activation *via* CD36/PPAR-γ pathway. Thirdly, Sg.LTA could induce a negative regulator that interferes with TLR2 signaling pathway in DCs. In fact, *Enterococcus faecalis* LTA decreased IL-8 production in human periodontal ligament cells stimulated with lipopolysaccharide (LPS) of oral pathogens, *Aggregatibacter actinomycetemcomitans* and *Porphyromonas gingivalis*, through induction of interleukin-1 receptor-associated kinase-M (28, 49).

In the current study, we found that Δ*ltaS* HKSG-sensitized DCs showed enhanced expression of co-inhibitory molecules, PD-L1 and PD-L2, compared with DCs sensitized with wild-type HKSG. In fact, enhanced expression of PD-L1 and PD-L2 seems to be one of the general phenomena which are frequently observed at the maturation of DCs (23, 50, 51). Nevertheless, the higher PD-L1 expression on Δ*ltaS* HKSG*-*pulsed DCs may possibly elicit reduced proliferation and activation of T cells because interaction between DCs and T cells through PD-L/PD-1 axis is known to suppress T cell proliferation and activation (52). However, our current study demonstrated that autologous T cell proliferation and activation were more potently induced by DCs sensitized with *ΔltaS* HKSG than those sensitized with the wild-type HKSG. To clearly figure it out, we further examined PD-1 expression on CD3^+^ T cells co-cultured with DCs pulsed with wild-type or *ΔltaS* HKSG. As shown in **Supplementary Figure 4**, T cells co-cultured with DCs sensitized with *ΔltaS* HKSG showed relatively lower PD-1 expression than those co-cultured with DCs sensitized with the wild-type HKSG. Besides, IL-12, which is considered as a key cytokine for T cell stimulation, was more potently induced by DCs sensitized with *ΔltaS* HKSG than those sensitized with wild-type HKSG. Therefore, high IL-12 production with low PD-1 expression on T cells co-cultured with *ΔltaS* HKSG-pulsed DCs is supposed to elicit T cell activation despite the increased expression of PD-L1 and PD-L2.

Most oral streptococci, including *S. oralis*, *S. sanguinis*, and *S. mutans* as well as *S. gordonii*, can cause opportunistic infections such as infective endocarditis (53, 54). For successful survival in the host, bacteria need to colonize host tissues and develop strategies for either developing a symbiotic relationship with the host or concealing themselves to evade the host defense system. Thus, the production of LTA might be a wise strategy for bacteria to survive in the host because LTA inhibits phagocytic activity of host immune cells (55) and preferentially induces anti-inflammatory signals (48, 56). On the other hand, LTA could interfere with the establishment of host immune responses during the bacterial infectious condition by antagonizing the B cell proliferation induced by LPS (57). Remarkably, LTA was reported to be rich in the septum and to be actively released at the bacterial division (58, 59). We suggest that membrane-anchored and/or released LTA of *S. gordonii* could interfere with sufficient activation of DCs and DC-mediated adaptive immune responses.

## Data availability statement

The original contributions presented in the study are included in the article/**Supplementary Material**. Further inquiries can be directed to the corresponding author.

## Ethics statement

All experiments using human blood were conducted with the approval of the Institutional Review Board of Seoul National University (S-D20180029).

## Author contributions

SKK, JI, HSS, and SHH designed research. SKK, EBK, DL, and JI carried out experiments. SKK, EBK, JI, DL, and SHH analyzed and interpreted data. SKK, JI, DL, HSS, C-HY and SHH prepared and reviewed the manuscript.
