# Supplementary figures and images for: Lipoteichoic acid of Streptococcus gordonii as a negative regulator of human dendritic cell activation
Source: Front Immunol. 2023 Mar 28;14:1056949. doi: 10.3389/fimmu.2023.1056949 (PMC10086370; doi:10.3389/fimmu.2023.1056949)

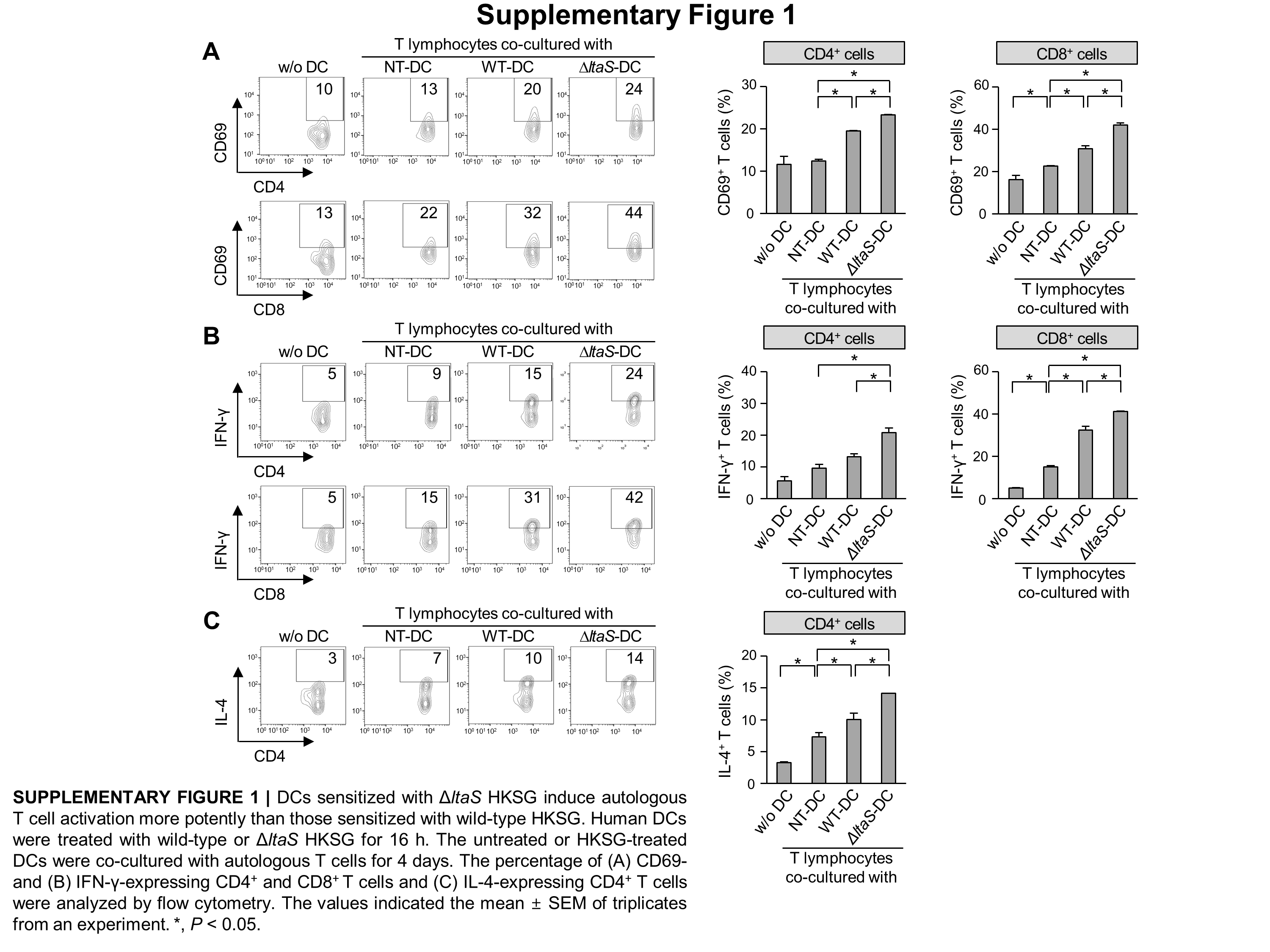

Supplement: Supplementary file 1 [file Image_1.tif]

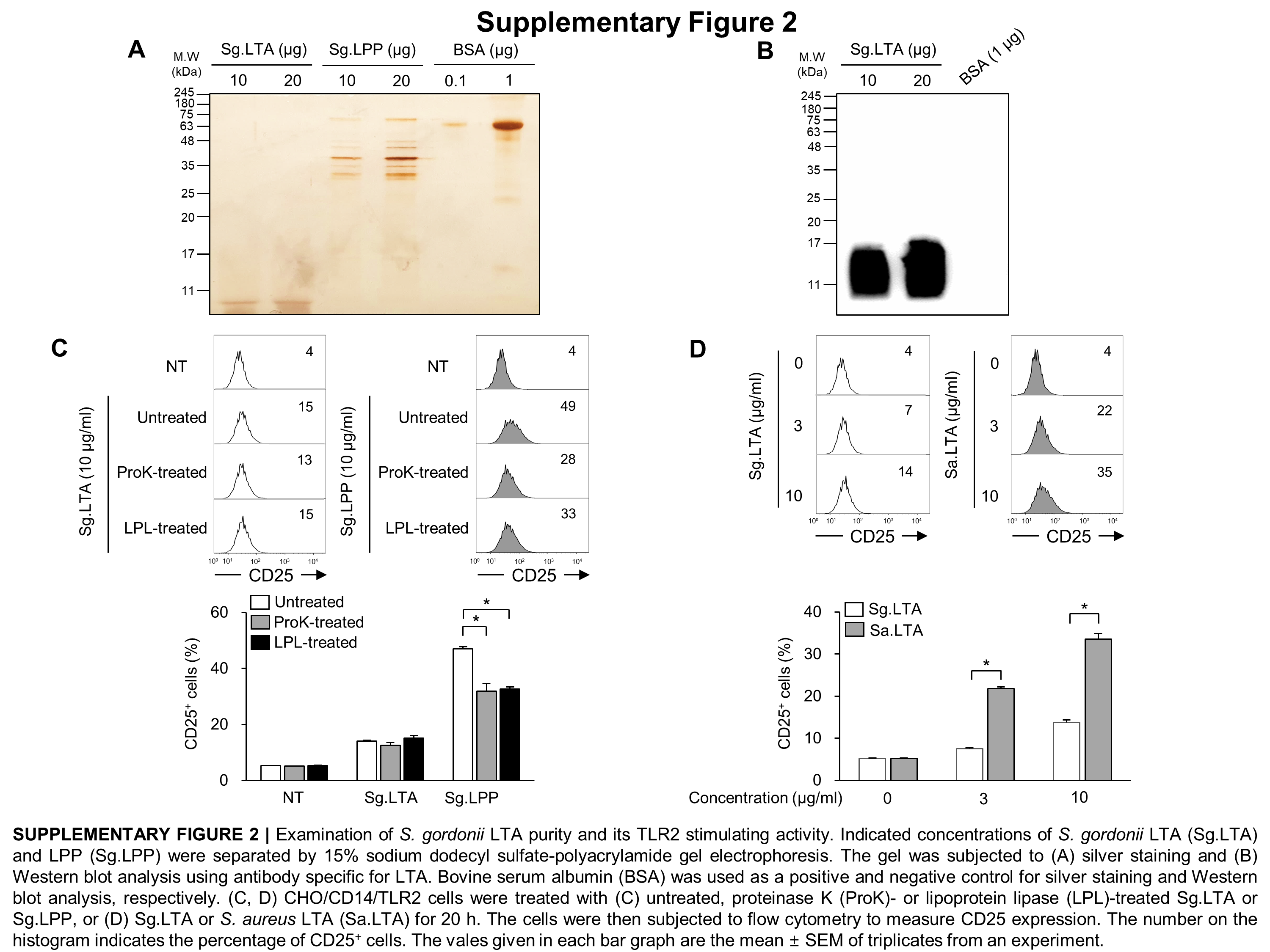

Supplement: Supplementary file 2 [file Image_2.tif]

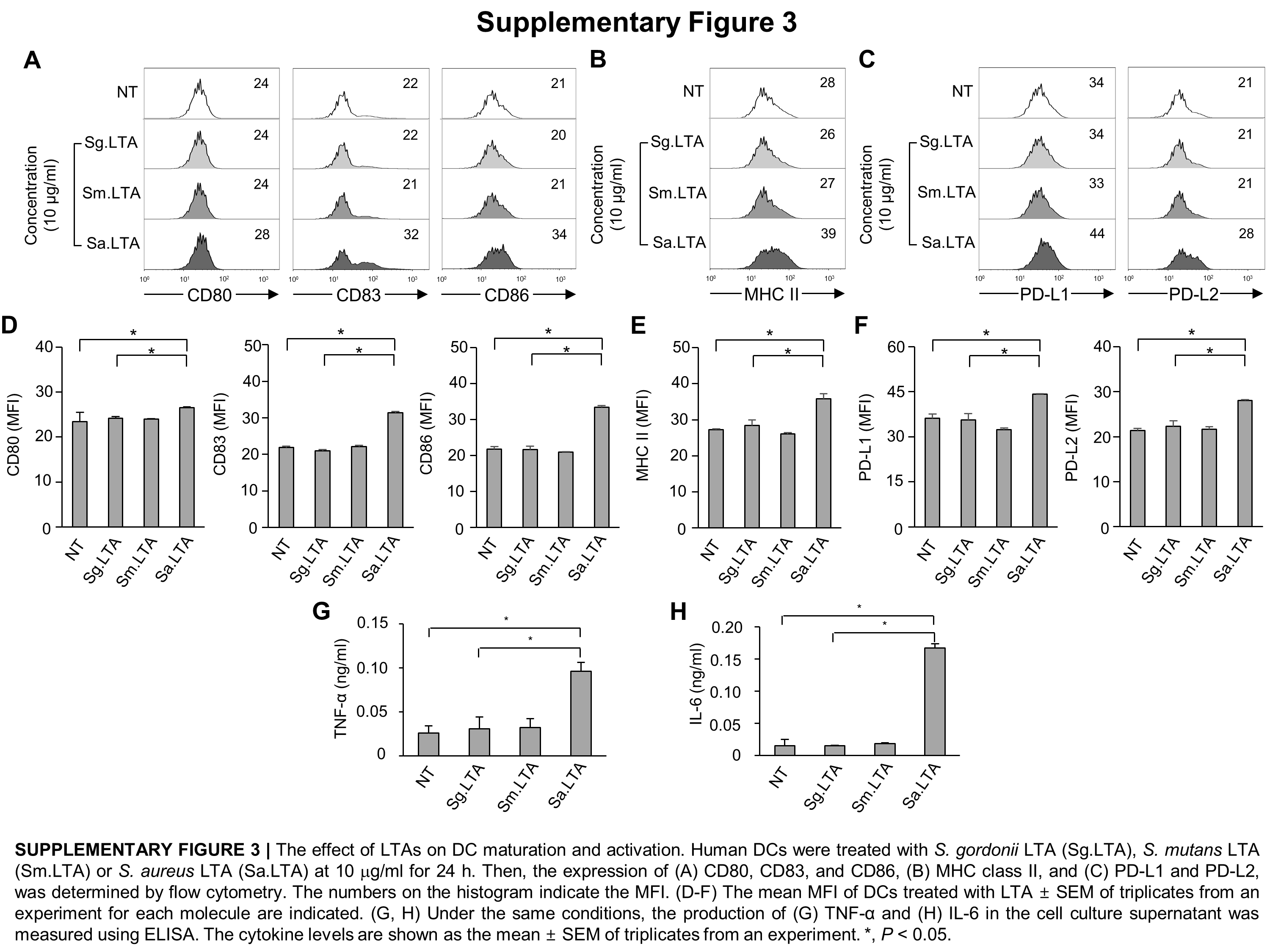

Supplement: Supplementary file 3 [file Image_3.tif]

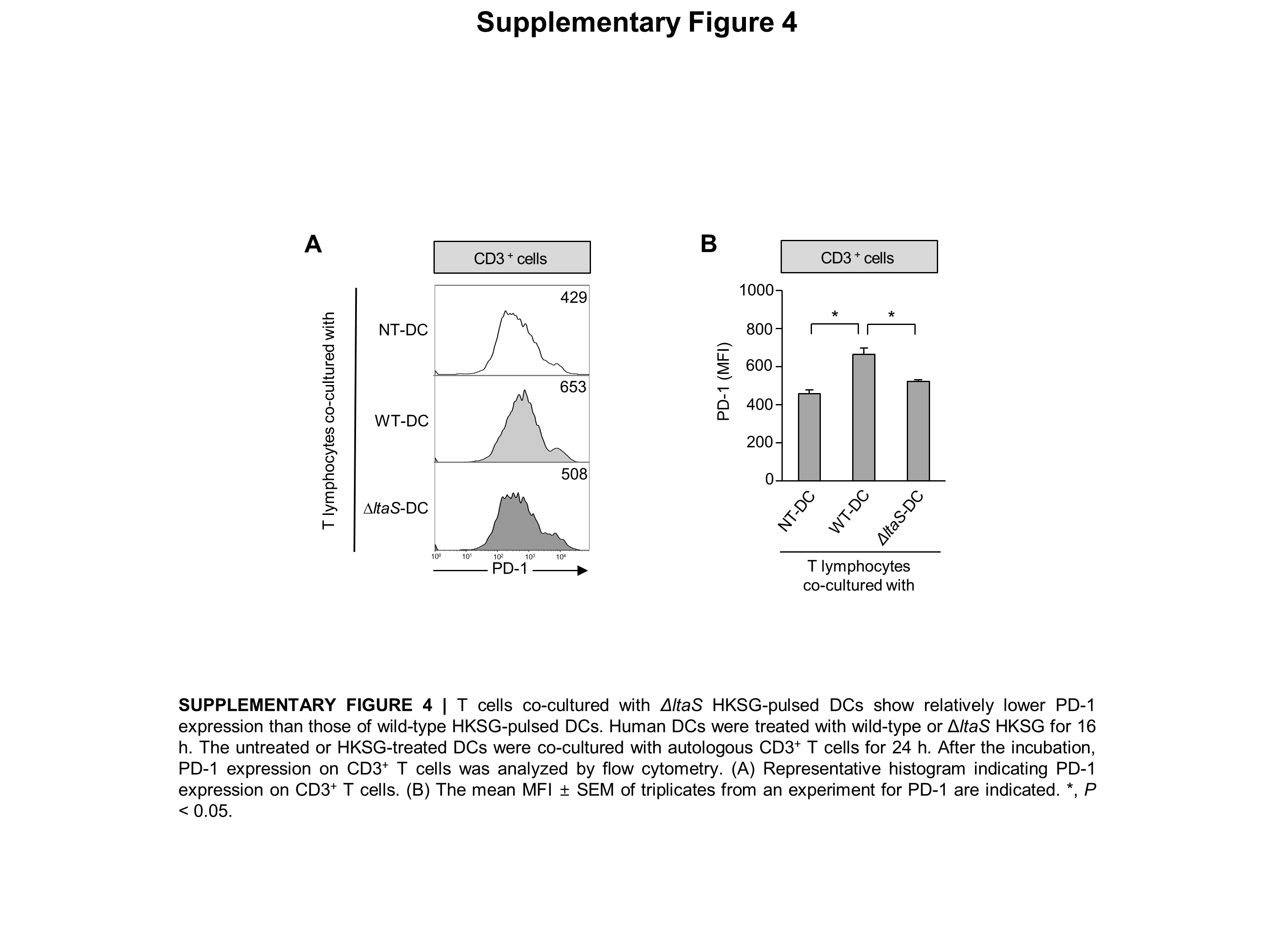

Supplement: Supplementary file 4 [file Image_4.tif]
